# The Use of Spray-Dried Mn_3_O_4_/C Composites as Electrocatalysts for Li–O_2_ Batteries

**DOI:** 10.3390/nano6110203

**Published:** 2016-11-07

**Authors:** Hong-Kai Yang, Chih-Chun Chin, Jenn-Shing Chen

**Affiliations:** Department of Applied Chemistry, National University of Kaohsiung, Kaohsiung City 81148, Taiwan; hong-kai84@hotmail.com (H.-K.Y.); ccchin17@gmail.com (C.-C.C.)

**Keywords:** Mn_3_O_4_/C, cathode, lithium-oxygen battery, rotating ring-disk electrode

## Abstract

The electrocatalytic activities of Mn_3_O_4_/C composites are studied in lithium–oxygen (Li–O_2_) batteries as cathode catalysts. The Mn_3_O_4_/C composites are fabricated using ultrasonic spray pyrolysis (USP) with organic surfactants as the carbon sources. The physical and electrochemical performance of the composites is characterized by X-ray diffraction, scanning electron microscopy, particle size analysis, Brunauer–Emmett–Teller (BET) measurements, elemental analysis, galvanostatic charge–discharge methods and rotating ring-disk electrode (RRDE) measurements. The electrochemical tests demonstrate that the Mn_3_O_4_/C composite that is prepared using Trition X-114 (TX114) surfactant has higher activity as a bi-functional catalyst and delivers better oxygen reduction reaction (ORR) and oxygen evolution reaction (OER) catalytic performance in Li–O_2_ batteries because there is a larger surface area and particles are homogeneous with a meso/macro porous structure. The rate constant (*k_f_*) for the production of superoxide radical (O_2_^•^^−^) and the propylene carbonate (PC)-electrolyte decomposition rate constant (*k*) for M_3_O_4_/C and Super P electrodes are measured using RRDE experiments and analysis in the 0.1 M tetrabutylammonium hexafluorophosphate (TBAPF_6_)/PC electrolyte. The results show that TX114 has higher electrocatalytic activity for the first step of ORR to generate O_2_^•^^−^ and produces a faster PC-electrolyte decomposition rate.

## 1. Introduction

Rechargeable lithium–oxygen (Li–O_2_) batteries are very efficient energy-storage devices and are used as power sources for electric vehicles (EV) and hybrid electric vehicles (HEV) because of their low cost, environmentally benign effects and high theoretical energy density (~3500 Wh·kg^−1^), which is almost nine times higher than that of current Li-ion batteries (~400 Wh·kg^−1^) [1,2,3,4]. Despite these favorable characteristics, their practical applications have still been hampered in the past decade because of their limited rate capability, poor cycling stability due to the instability of the electrode and electrolyte, and low round-trip efficiency induced by excessive polarization, resulting in a wide charge–discharge voltage gap [1,2,3,4,5,6,7,8]. These critical problems are predominantly caused by the O_2_ cathode.

Many studies [1,4,7,8,9,10,11] have shown that the electrochemical performance of Li–O_2_ batteries depends on many factors, such as the nature and microstructure of the O_2_ electrode, the formulation of the electrolyte (especially, the composition of the solvent), the possible presence of reactive contaminants (e.g., trace water) and the choice of catalysts. In order to enhance the properties of rechargeable Li–O_2_ batteries, most studies have focused on the electrolyte formula, choice, and microstructure design of the O_2_ electrode/electrocatalyst, and optimization of the operating parameters [1,3,8]. Because there is poor electrochemical reversibility on the oxygen cathode side, a cathode catalyst that is highly active and has good chemical stability is necessary for good battery performance [5,12]. Therefore, a bi-functional cathode catalyst that facilitates the complete reversibility of oxygen reduction reactions (ORRs) and oxygen evolution reactions (OERs) at low polarization in Li–O_2_ batteries is required. Several potential electrocatalysts that promote ORRs and OERs in Li–O_2_ batteries have recently been proposed, including nitrogen-doped carbon, metal oxides, metal nitrides, precious and nonprecious metals, etc. [1,2,6,11,13,14,15,16,17]. Among metal oxides, manganese oxide is a catalyst material of great interest owing to its low cost, environmental friendliness, abundance, and electrocatalytic activity for ORRs in Li–O_2_ batteries [1,6,16,18]. Carbon-supported manganese oxide (MnO*_x_*, Mn_3_O_4_, MnOOH et al.), which combine the good catalytic performance of manganese oxide with the optimized morphology and size of carbon materials, is a preferred electrocatalyst for ORRs and OERs in Li–O_2_ batteries [1,19,20,21]. Therefore, this study of Li–O_2_ batteries focuses on Mn_3_O_4_/C catalysts.

Ultrasonic spray pyrolysis (USP) is eminently suitable for the fabrication of heat-treated solid-state materials because the manufacturing process is a one-step process and produces spherical and uniform particles with a particle size that can be controlled. This study uses the USP method to prepare homogeneous spherical Mn_3_O_4_/C particles and uses various organic surfactants, such as Trition^®^ X-114 (TX114), Pluronic^®^ F-127 (F127) and Pluronic^®^ P-123 (P123), as structure-directing agents and residual carbon sources. The aim of this work is to develop a new heat-treatment method for the synthesis of high-surface-area Mn_3_O_4_/C catalysts and demonstrate the significant effect of morphology on the electrocatalytic performance of cathode catalysts for Li–O_2_ batteries. We present a detailed study of the Li–O_2_ electrochemistry of the Mn_3_O_4_/C material, using an electrolyte of 1 M LiPF_6_ in a propylene carbonate (PC) solvent. The PC solvent is used because it was used in many of the initial studies on Li–O_2_ batteries, in spite of its poor stability. Although there have been many studies of the use of Mn_3_O_4_/C materials in Li–O_2_ batteries, few studies have examined the poor stability of the electrolyte because of its reaction with the superoxide radical (O_2_^•^^−^) that is produced upon the discharge at the Mn_3_O_4_/C electrode. Therefore, as the reported in our early study [6], aspects of the PC-based electrolyte reaction against O_2_^•^^−^ and the related kinetic information of O_2_^•^^−^ in the Mn_3_O_4_/C electrode are explored by studying rotating ring-disk electrode (RRDE) experiments and using a lithium-free non aqueous electrolyte because of the stability of the intermediate O_2_^•^^−^. The O_2_ solubility, diffusion rates of O_2_ and superoxide radical (O_2_^•^^−^) coefficients ($D_{O_{2}}$ and $D_{O_{2}^{•-}}$), rate constant (*k_f_*) for the production of O_2_^•^^−^ and PC-electrolyte decomposition rate constant (*k*) of the as-prepared Mn_3_O_4_/C electrode are measured.

## 2. Experimental Methods

**Figure 1:**
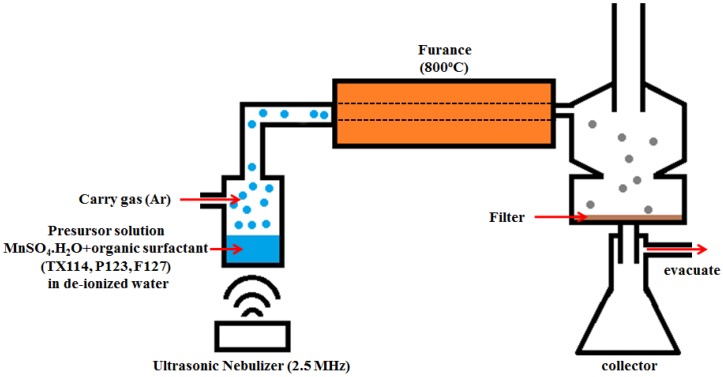
A schematic diagram of the experimental apparatus for ultrasonic spray pyrolysis (USP).

Mn_3_O_4_/C composites were prepared using ultrasonic spray pyrolysis (USP) with manganese sulfate as the manganese ion precursors and organic surfactants as the structure-directing agents and carbon sources. The schematic diagram for the USP experimental apparatus is given in Figure 1. A humidifier ultrasonically nebulizes a precursor solution to form micrometer-size aerosol droplets. The aerosol droplets are then carried by Ar gas into a furnace, where the droplets evaporate and the precursor decomposes. The final solid products were collected by filtration. A precursor solution was prepared by dissolving 90 wt % manganese sulfate monohydrate (MnSO_4_·H_2_O, Sigma Aldrich, St. Louis, MO, USA) and 10 wt % organic surfactants into deionized water, to achieve a 0.5 M concentration of MnSO_4_. Three different organic surfactants were used: TX 114 (Trition^®^ X-114, Sigma Aldrich, St. Louis, MO, USA), F127 (Pluronic^®^ F-127, Sigma Aldrich, St. Louis, MO, USA) and P123 (Pluronic^®^ P-123, Sigma Aldrich, St. Louis, MO, USA). The homogeneous precursor solution was obtained by ultrasonic treatment and was converted into an aerosol using a 2.4 MHz ultrasonic nebulizer (Model WB-P2424FX, Whirl Best, Taoyuan City, Taiwan), which produces small droplets. The aerosol droplets were carried into a heated quartz tube (diameter: 25 mm, length: 30 cm) by a continuous high-purity Ar flow (500 mL·min^−1^) for sulfate pyrolysis and microsphere solidification. The quartz tube was installed inside a split-hinge tube furnace, which was maintained at 800 °C. The solid particles that formed were collected in a vacuum filter at the other end of quartz tube. The final microsphere Mn_3_O_4_/C composites were isolated on 450 nm filter paper, washed sequentially with absolute ethanol and purified water several times, and dried at 110 °C for 6 h.

A Rigaku-D/MaX-2550 diffractometer (Rigaku, Tokyo, Japan) with Cu K_α_ radiation (λ = 1.54 Å) was used to obtain X-ray diffraction (XRD) patterns for the samples. The morphology of the sample was observed using a scanning electron microscope (SEM, Hitachi S-3400 Hitachi Limited, Tokyo, Japan). The Brunauer-Emmett-Teller (BET) method was used to measure the specific surface area of the powders (ASAP2020). Particle size analysis (PSA) used a Malvern particle size analyzer (Zetasizer Nano ZS, Malvern Instruments Ltd, Malvern, UK). The residual carbon content of the samples was determined using an automatic elemental analyzer (EA, Elementar vario EL III Elementar Analysensysteme GmbH, Hanau, Germany).

For electrochemical tests, the Mn_3_O_4_/C electrodes were prepared by wet coating. They were made from as-prepared Mn_3_O_4_/C composites with super P and a poly(vinylidene difluoride) (PVDF) binder (MKB-212C, Elf Atochem, Atofina, Serquigny, France) in a weight ratio of 64:16:20. The Mn_3_O_4_/C composites and super P were first added to a solution of PVDF in *N*-methyl-2-pyrrolidone (NMP, Riedel-deHaen, Seelze, Germany). The mixture was stirred for 20 min at room temperature using a magnetic stir bar, and then for 5 min using a turbine at 2000 rpm in order to produce a slurry with an appropriate viscosity. The resulting slurry was coated onto a piece of separator (Celgard 2400, Charlotte, NC, USA) and dried at 60 °C under vacuum for 12 h. The coating had a thickness of ~100 μm with an active material mass loading of 8 ± 1 mg·cm^−2^. The quantity of active materials on the electrodes was kept constant. The electrodes were dried overnight at 60 °C under vacuum, before being transferred into an argon-filled glove box for cell assembly. The Li–O_2_ test cell (EQ-STC-LI-AIR, MTI Corporation, Richmond, CA, USA) was constructed using lithium metal as the negative electrode and the Mn_3_O_4_/C electrode as the positive electrode. A solution of 1 M LiPF_6_ in a PC solvent was used as the electrolyte for all cells. After assembly, the test cell was removed from the Ar-filled glove box and attached to a gas manifold that was constantly purged with dry O_2_. The electrochemical tests were performed after the cell has been flushed with O_2_ for 6 h. The cells were galvanoststically cycled using a BAT-750B (Acu Tech System, Taipei, Taiwan) at a constant current of 0.2 mA·cm^−2^ with a voltage of 2.0–4.5 V vs. Li/Li^+^ at room temperature.

For RRDE experiments, an RRDE system (AFMT134DCPTT, Pine Research Instruments, Durham, NC, USA) with interchangeable disks consists of a 5 mm diameter glassy carbon electrode and a Pt ring electrode (1 mm width) with a 0.5 mm gap between them. The collection efficiency for this geometry is 0.24. The rotating ring-disk assembly was operated on a Pine AFMSRX rotator and CH705 Bipotentiostat (CH Instruments, Austin, TX, USA) with a computerized interface. Experiments were conducted using a three-electrode cell containing 10 mL of the electrolyte of interest. The cell was assembled in a dry Ar-filled atoms bag (Sigma-Aldrich Z108450, ST. Louis, MO, USA). The counter electrode was a Li foil that was connected to an Ag wire and isolated by a layer of Celgard 2400 separator to prevent convective oxygen transport to the electrode. The Ag/Ag^+^ reference electrode consisted of an Ag wire that was immersed into 0.1 M AgNO_3_ in CH_3_CN and sealed with a vycor frit at its tip. All potentials in this study are referenced to the Li/Li^+^ potential scale (volts vs. Li^+^/Li or V_Li+_), obtained by calibration of the reference electrode against a fresh lithium wire before the experiments (0 V_Li_ = −3.46 ± 0.01 V vs. Ag/Ag^+^). The working electrode consisted of a catalyst-covered glassy carbon disk and was immersed into the Ar or O_2_-purged electrolyte for 30 min before each experiment. Prior to the RRDE measurements, alternating current (AC) impedance measurements determined the uncompensated ohmic drop between the working and the reference electrodes. A 10 mV perturbation (0.1 MHz to 10 MHz) was applied to the open circuit. The IR-correction to remove ohmic losses used the total cell resistance of ~293 Ω, as measured by AC impedance. The capacitive-corrected ORR currents were calculated by subtracting the current measured under Ar from that obtained in pure O_2_ under identical scan rates, rotation speeds and catalyst loadings.

## 3. Results and Discussion

**Figure 2:**
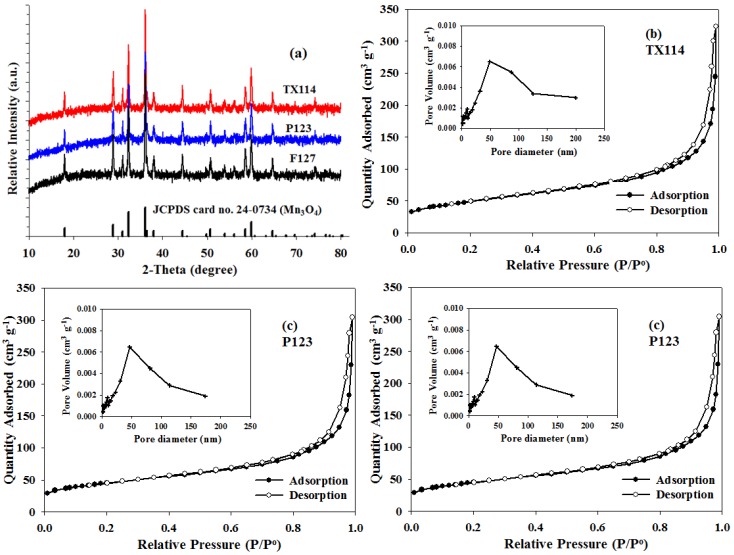
(**a**) X-ray diffraction (XRD) patterns for a theoretical pattern, Pluronic F-127 (F127), Pluronic P-123 (P123), and Trition X-114 (TX114) and N_2_ adsorption-desorption isotherms and the pore-size distribution plots (insert) for (**b**) TX114; (**c**) P123; and (**d**) F127.

The phase composition and the crystal structure of the as-prepared composites were determined using the XRD patterns that are shown in Figure 2a. In Figure 2a, all peaks are identified as pure and well-crystallized Mn_3_O_4_ phase (JCPDS 24-0734), with a hausmannite-type tetragonal structure that is indexed to the I4_1_/*amd* soace group. The XRD curves do not show any evidence of the formation of crystalline or amorphous carbon. It appears that when using different organic surfactants as a carbon source, all of the final products probably remain amorphous or as low crystalline carbon.

Nitrogen adsorption-desorption isothermal measurements were performed to determine the pore structure of the Mn_3_O_4_/C composite. Figure 2b–d shows that all of the as-prepared Mn_3_O_4_/C samples exhibit a typical type-IV N_2_ sorption isotherms with distinct H3-type hysteresis loops at a high relative pressure between 0.8 and 1.0, indicating the characteristic of macroporous and mesoporous materials. The Barrett–Joyner–Halenda (BJH) pore size distributions for all samples are shown in the inserts of Figure 2b–d along with a peak that is centered at 50 nm. The minor broad distribution peak that ranges between 100 and 200 nm corresponds to the macropore region and is probably a result of the space between aggregated particles. Many studies [13,22,23] have shown that the wide variation in meso/macro pore size increases the cell capacity of Li–O_2_ batteries because it results in a large electrolyte/electrode contact area and favorable distribution of the discharge products, such as lithium peroxide, in the cathode discharge. The BET analysis shows respective specific surface areas of about 28.9, 23.3 and 23.0 m^3^·g^−^^1^ for the TX114, P123 and F127.

**Figure 3:**
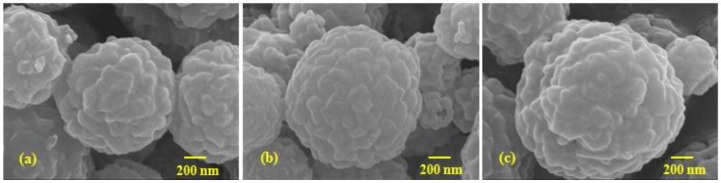
Scanning electron microscope (SEM) images for Mn_3_O_4_/C composites that use (**a**) TX114; (**b**) P123; and (**c**) F127.

The morphology of the as-prepared Mn_3_O_4_/C composites was observed by SEM, as shown in Figure 3. The SEM images of the Mn_3_O_4_/C composite show that the shape of the Mn_3_O_4_/C powder is close to spherical and the size is narrowly distributed between 0.9 and 1.3 μm, although a few agglomerates exist. The mean particle sizes of the TX114, P123 and F127, as determined by PSA, are about 0.86, 1.04 and 1.51 μm, respectively. These values are in good agreement with the measurement from SEM (Figure 3). Table 1 shows the residual carbon content, the particle size and the BET surface area for all of the as-prepared Mn_3_O_4_/C composites, using TX114, P123 and F127 surfactants. In order to ensure that there were equal amounts of residual carbon in the composites, the final content of the residual carbon of all of the samples was maintained at approximately 2–3 wt %. The data in Table 1 demonstrates that the composite from TX114 has a smaller particle size, which results in a larger surface area. The particle size increases, which is consistent with the decrease in the specific surface area, from 28.9, 23.3 and 23.0 m^2^·g^−1^, for the TX114, P123 and F127, respectively.

Due to the study of the electrocatalytic activity of the as-prepared Mn_3_O_4_/C samples, the following discussion of the electrochemical tests makes comparisons between Super-P carbon (SP) and as-prepared Mn_3_O_4_/C materials. Mn_3_O_4_ is a highly active ORR catalyst and has recently been used as an O_2_ cathode catalyst in Li–O_2_ batteries [5,19,21]. Therefore, it is essential to determine the kinetics of ORR for the as-prepared Mn_3_O_4_/C composites. The rotating ring disk electrode (RRDE) technique was used to determine the kinetics of ORR, since the ORR current is strongly affected by hydrodynamic conditions. In an early paper by the authors [6], the kinetics of ORR for a MnO_2_/C composite was studied and the O_2_ solubility, the diffusion rates of O_2_ and O_2_^•^^−^ coefficients ($D_{O_{2}}$ and $D_{O_{2}^{•-}}$), the rate constant (*k_f_*) for producing O_2_^•^^−^ and the propylene carbonate (PC)-electrolyte decomposition rate constant (*k*) for the MnO_2_/C material and SP were measured using RRDE experiments in a 0.1 M TBAPF_6_/PC electrolyte. This study uses similar RRDE experiments to determine the kinetics of ORR for the as-prepared Mn_3_O_4_/C composites. The O_2_^•^^−^ produced in the first step of the ORR, when the Li–O_2_ battery discharges:

(1) $$O_{2}+e^{-}\overset{k_{f}}{\to }{O_{2}}^{•-}$$

In the PC-based electrolyte, the ethereal carbon atom in PC suffers from nucleophilic attacks by O_2_^•^^−^, which yields carbonate, acetate, and formate species among others, according to Equation (2) [24]:

(2) $$PC+{O_{2}}^{•-}\;\overset{k}{\to }\;{{CO}_{3}}^{2-},\;{\mathrm{HCOO}}^{-},\;{{CH}_{3}\mathrm{COO}}^{-}$$

The reaction rate constant, *k_f_*, is evaluated using the Koutecky-Levich (K-L) equation for a first order reaction, as follows:

(3) $$\frac{1}{i}=\frac{1}{i_{k}}+\frac{1}{i_{d}}\;$$

(4) $$i_{k}=nFk_{f}C_{O_{2}}$$

(5) $$i_{d}=0.62nFD_{O_{2}}^{2/3}\nu^{-1/6}C_{O_{2}}\omega^{1/2}$$

where *i_k_* and *i_d_* respectively represent the kinetics and the diffusion limiting current density (A·m^−^^2^), *n* is the number of electrons that are exchanged in the electrochemical reaction, *F* is Faraday’s constant (96485 C·mol^−^^1^), *k_f_* is the rate constant for reaction 1, $D_{O_{2}}$ is the diffusion coefficient of O_2_ in the solution, *υ* is the kinematic viscosity, ω is the angular frequency of the rotation, and $C_{O_{2}}$ is the saturation concentration of O_2_ in the solution. The stability of an electrolyte against O_2_^•^^−^ is defined by the rate constant (*k*) for Equation (2) and is quantified using the RRDE voltammetry [6,24]. The O_2_^•^^−^ that is produced at the disk electrode in Equation (1) and the amount of O_2_^•^^−^ are quantified at the ring electrode using the decrease in the collection efficiency, *N_k_*, for O_2_^•^^−^ at the ring electrode as the transient time increases. The correlation with the collection efficiency is the absolute ratio of the ring and disk currents and is characterized by the following equation [6,24,25]:

(6) $$N_{k}=-\frac{i_{ring}}{i_{disk}}=N_{geometerical}-\beta^{\frac{2}{3}}\left(1-UA_{1}^{-1}\right)+\frac{1}{2}A_{1}^{-1}A_{2}^{2}\kappa^{2}U\beta^{\frac{4}{3}}-2A_{2}\kappa^{2}T_{2}$$

where *A*_1_ = 1.288, *A*_2_ = 0.643ν^1/6^$D_{O_{2}^{•-}}$^1/3^, β = 3ln(*r*_3_/*r*_2_), *U* = *k*^−1^ tanh(*A*_1_*k*) and *T*_2_ = 0.718 ln(*r*_2_/*r*_1_), whereby *r*_1_, *r*_2_, and *r*_3_ refer to the radius of the disk and internal and external ring radii, respectively; ν is the kinematic viscosity; ω is the rotation rate; *k* is the rate constant for reaction 2; and $D_{O_{2}^{•-}}$ is the diffusion coefficient of O_2_^•^^−^. *N_geometrical_* is the geometrical collection efficiency of the RRDE corresponding to the fraction of a species electrochemically generated at the disk. This species is detected at the ring due to the lack of side-reactions with the electrolyte. From the measurement of *N_k_* at a given rotation rate (ω), the rate constant (*k*) can be calculated by Equation (6).

**Figure 4:**
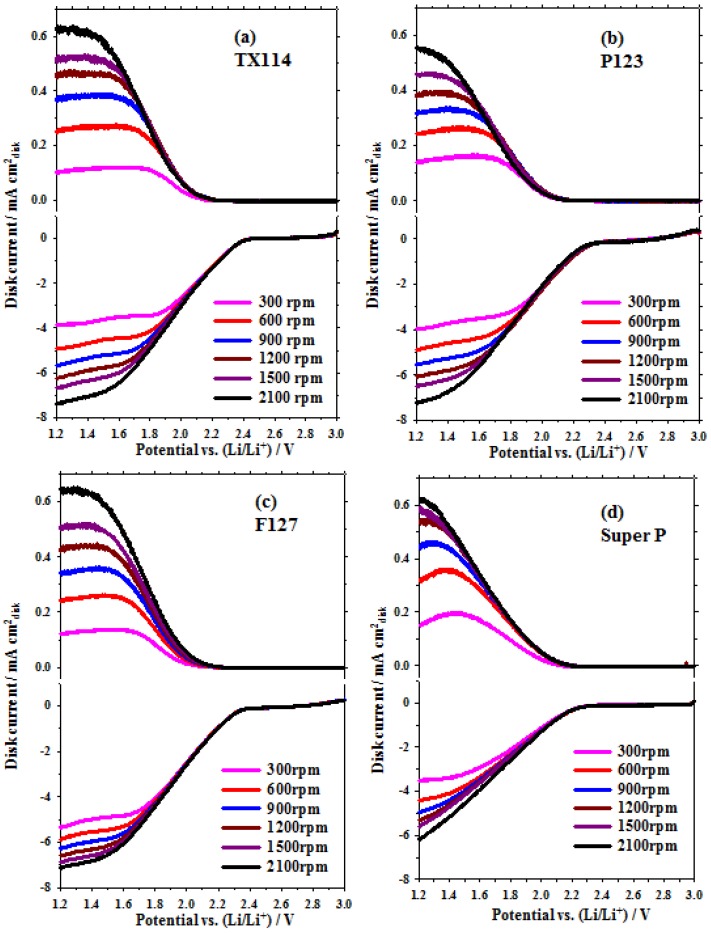
RRDE profiles for Mn_3_O_4_/C composites and Super P (SP), recorded at 50 mV·s^−1^ in an O_2_-saturated 0.1 M TBAPF_6_/propylene carbonate (PC) solution, at rotation rates between 300 and 2100 rpm (the Pt ring is maintained at 2.6 V_Li_: (**a**) TX114; (**b**) P123; (**c**) F127; and (**d**) Super P.

**Figure 5:**
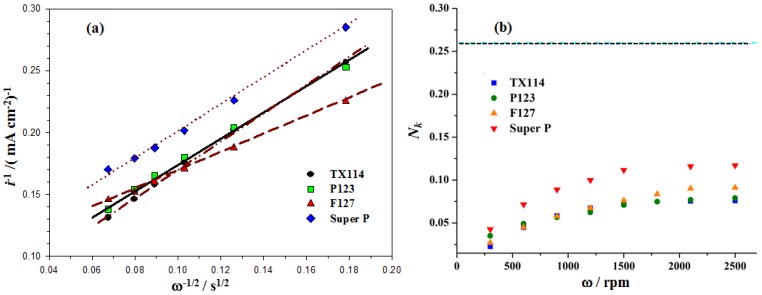
(**a**) Koutecky-Levich (K-L) plots for TX114, P123, F127 and Super P (SP). These are derived from the disc current values at 1.3 V*_Li_*; (**b**) The evolution of the absolute ratio between the ring and the disk current (*N_k_*) and the electrode rotation rate (ω) for TX114, P123, F127 and Super P (SP), recorded at 50 mV·s^−1^ in an O_2_-saturated 0.1 M TBAPF_6_/PC solution (see Figure 4).

Prior to estimating the value of rate constants (*k_f_* and *k*), the kinematic viscosity (ν) of the electrolyte and the diffusion coefficients of O_2_ and O_2_^•^^−^ ($D_{O_{2}}$ and $D_{O_{2}^{•-}}$) must be quantified. These are listed in Table 2. The estimation of the values in Table 2 is reported in an early paper by the authors [6]. Figure 4 shows the RRDE profiles for the Mn_3_O_4_/C and SP samples that are coated on the disk electrode. The disk and ring currents were recorded in an O_2_-saturated 0.1 M TBAPF_6_/PC solution at rotation rates between 300 and 2100 rpm. The Pt ring was maintained at 2.6 V_Li_ . The K-L plots for the disc current values at 1.30 V_Li_ (see Figure 4) show the expected linear relationship between the inverse of the limiting current (*i_d_*) and ω*^−^*^0^^.5^ (see Equation (5)). The rate constant for the production of O_2_^•^^−^, *k_f_* for the GC and the MnO_2_/C-GC electrodes is obtained by linearly fitting the K-L plots of *i*^−^^1^ vs. ω*^−^*^0.5^ (see Equation (5)) as shown in Figure 5a. The values of *k_f_* for all of the as-prepared Mn_3_O_4_/C composites that use TX114, P123 and F127 surfactants and SP electrodes are 3.7 × 10^−^^2^ cm·s^−^^1^, 2.9 × 10^−^^2^ cm·s^−^^1^, 2.3 × 10^−^^2^ cm·s^−^^1^ and 2.1 × 10^−^^2^ cm·s^−^^1^, respectively. This result indicates that the Mn_3_O_4_/C cathode catalyst has a larger *k_f_* value than SP, because there is a higher electrocatalytic activity for the first step of the ORR (see Equation (1)), so a higher concentration of O_2_^•^^−^ is produced. The TX114 sample has the smallest particle size so it has the largest specific surface area of all of the samples. This results in the highest activity for the ORR in the O_2_ electrodes.

The RRDE profiles show that the ring current increases as the rotation rate increases because the shorter transient time at higher rotation rates reduces the reaction time between O_2_^•^^−^ and the PC electrolyte so a higher concentration of superoxide radical is oxidized at the ring, as shown in Figure 4. Figure 5b shows that the value of *N_k_* increases as the rotation rate increases and the individual constant value is close to ω = 2500 rpm for all samples. The values for *N_k_* at ω = 2500 rpm are about 0.07, 008, 0.09 and 0.1 for TX114, P123, F127 and SP, respectively. The PC-electrolyte decomposition rate constant (*k*) for different samples is evaluated using Equation (6), using the *N_k_* value at a rotation rate of 2500 rpm and the kinematic viscosity (ν) and $D_{O_{2}^{•-}}$ values that are listed in Table 2. Table 3 shows the rate constant for the production of O_2_^•^^−^, *k_f_*, and the PC-electrolyte decomposition rate constant, *k*, for the TX114, P123, F127 and SP electrodes. It is seen that the *k* values for the Mn_3_O_4_/C-GC that is prepared using different surfactants are larger than that for the SP. These results also show that TX114 is the most active surfactant for the first step of the ORR (the largest rate constant; *k_f_*). It produces the highest concentration of O_2_^•^^−^, so the PC-electrolyte decomposes fastest because it is attacked by a large amount of O_2_^•^^−^. A similar result was reported in an earlier study by the authors [6].

**Figure 6:**
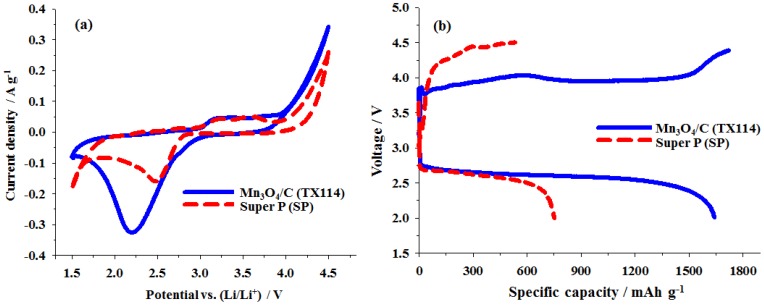
(**a**) CVs recorded at a scanning rate of 2 mV·s^−1^ for TX114 and Super-P (SP); (**b**) The initial charge–discharge profiles for TX114 and SP at a current density of 0.2 mA·cm^−2^.

To better determine the electrocatalytic activity of the electrodes, cyclic voltammetry (CV) and charge–discharge voltage measurements were performed in the Li–O_2_ test cell to compare Mn_3_O_4_/C composite (prepared from TX114) and Super P carbon (SP). The CV plots for the O_2_ electrodes that were prepared using TX114 and SP cycled between 1.5 and 4.5 V at 2 mV·s^−1^ and for the 1 M LiPF_6_/PC electrolyte are shown in Figure 6a.

During discharge for a Li–O_2_ cell, a single cathodic peak appeared at ~2.6 V, indicative of one-step oxygen reduction to produce reaction products by ORR. Upon charging, another anodic peak appeared at ~3.3 V, indicative of decomposition reaction products by OER. The comparison of CV curves for Li–O_2_ cells with TX114 and SP cathodes were shown in Figure 6a. From the CV curves, the reduction peak voltage is shifted toward a positive voltage, exhibiting electrocatalytic activity in the ORR of both samples. However, the TX114 produces more positive onset reduction peak potential and a larger peak current, which clearly demonstrate that the electrocatalytic activity of TX114 is superior to that of SP. The onset oxidation peaks that appear in the CV curves are about 2.6 and 3.1 V for TX114 and SP, respectively. Therefore, TX114, with its lower onset oxidation peak, is more efficient for Li_2_O_2_ decomposition and has higher catalytic activity for the OER. The initial charge–discharge voltage profiles for both samples at the current density of 0.2 mA·cm^−2^ are shown in Figure 6b. The charge–discharge profiles for the TX114 electrode exhibit much lower charge overpotential than do those of the SP electrode, although the reduction of the total overpotential is only about 30%. The round-trip efficiencies of the Li–O_2_ batteries with a TX114 electrode were lower than those with the SP electrode. These results demonstrate that the Mn_3_O_4_/C composite can facilitate the complete reversibility of ORR and OER at low polarization for a Li–O_2_ battery. This finding is in good agreement with the results for the CV measurement. Obviously, the initial discharge capacity for the TX114 electrode reached a higher value of 1639 mAh·g^−1^ and the corresponding discharge plateau was up to 2.64 V. By contrast, the SP electrode delivered lower discharge capacity of 752 mAh·g^−1^ at the same current density, which may refer to its lower ORR catalytic activity.

**Figure 7:**
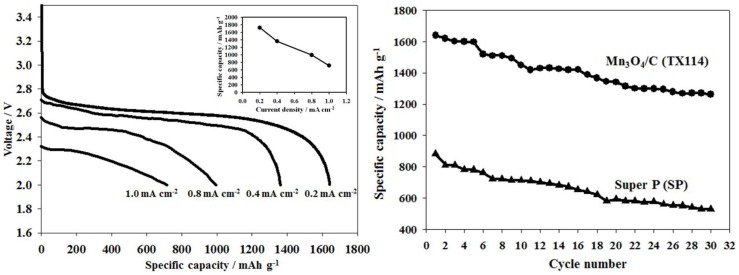
(**a**) Discharge curves at different current densities ranging from 0.2 mA·cm^−^^2^ to 1.0 mA·cm^−^^2^ for TX114. The insert is the discharge capacities at various current densities; (**b**) Cycle performance for TX114 and SP at a current density of 0.2 mA·cm^−2^.

Figure 7a shows the discharge curve of Li–O_2_ batteries utilizing the TX114 electrode at the different current densities of 0.2, 0.4, 0.8 and 1.0 mA·cm^−2^. With an increase of the discharge current density, the achieved capacity and cell potential were reduced, due to the internal resistance of the cells. The discharge capacities at a current density of 0.4, 0.8 and 1 mA·cm^−2^ shown in the insert of Figure 7a were found to be 1360, 994, 715 mAh·g^−1^, respectively. The cycling performances of Li–O_2_ batteries with TX114 and SP electrodes at a current density of 0.2 mA·cm^−2^ are shown in Figure 7b. As seen, the discharge capacity of the Li–O_2_ battery with the TX114 electrode preserved about 1263 mAh·g^−^^1^ after 30 cycles, and the capacity retention was about 77%, as opposed to only 530 mAh·g^−^^1^ discharge capacity (60%) for the SP electrode. These results indicate the superior cyclic stability of the TX114 electrode compared to the SP electrode. However, the capacity retention remained rather poor for both electrodes, with the discharge capacities dropping dramatically to below 80% after 30 cycles due to the continuous rise of the resistance. Until now, poor cycling stability has remained a significant challenge for Li–O_2_ cells. Many investigations [1,2,11,17,26,27,28] have shown that the biggest obstacle for cycling in Li–O_2_ cells is the decomposition of the electrolyte during the charge–discharge process. Our above RRDE results demonstrate that PC is a poor stable electrolyte solvent in Li–O_2_ battery due to its reaction with the superoxide radical at the O_2_ electrode, in agreement with the literature [1,11,17,24]. The above galvanostatic charge–discharge tests show that the discharge capacity of Mn_3_O_4_/C composite (TX114) was higher than that of our previous MnO_2_/C composite [6]. This indicates the superior electrocatalytic activity of Mn_3_O_4_/C composite compared to MnO_2_/C composite. Obviously, Mn_3_O_4_/C composite that uses TX114 has higher activity as a bi-functional catalyst and delivers better ORR and OER catalytic performance in Li–O_2_ batteries. Except PC, the choice of a novel electrolyte is very important, to avoid decomposition by O_2_^•^^−^ attack when applying an active catalyst on the cathode material in Li–O_2_ batteries. More detailed RRDE experiments and analysis will be performed in order to estimate the decomposition rates for various electrolytes with different bi-functional oxygen electrocatalysts.

## 4. Conclusions

Mn_3_O_4_/C catalyst materials have been synthesized using ultrasonic spray pyrolysis with various organic surfactants. SEM and BET analysis confirmed that the as-prepared Mn_3_O_4_/C composites consist of homogeneous, micro-spherical particles that have a meso/macro porous structure. The composite that uses a TX114 surfactant has a smaller particle size and larger specific surface area.

The rate constant for the production of O_2_^•^^−^ (*k_f_*) and the PC-electrolyte decomposition rate constant (*k*) for the TX114, P123, F127 and SP electrodes are measured by RRDE experiments in a 0.1 M TBAPF_6_/PC electrolyte. The results show that the Mn_3_O_4_/C composite that uses TX114 is the most active for the first step of the ORR (the largest rate constant; *k_f_*). It produces the highest concentration of O_2_^•^^−^, so the PC-electrolyte is decomposed fastest because it is attacked by a large amount of O_2_^•^^−^. The CV and galvanostatic charge–discharge tests demonstrate the TX114 has a higher activity as a bi-functional catalyst and delivers better ORR and OER catalytic performance in Li–O_2_ batteries. The choice of electrolyte is crucial when an active catalyst is used for the cathode material in Li–O_2_ batteries. In the near future, more detailed RRDE experiments and analysis will be performed in order to estimate the decomposition rates for various electrolytes, except PC. These results are relevant to the design of high-performance, rechargeable Li–O_2_ batteries.

## Figures and Tables

**Table 1:** A comparison of the residual carbon content, the particle size and the surface area of Mn_3_O_4_/C powders that use various surfactants.

Surfactants	Residual Carbon Content (wt %)	Particle Sizes (nm)	Brunauer–Emmett–Teller (BET) Surface Area (m^2^·g^−1^)
Trition X-114 (TX114)	2.56	855	26.9
Pluronic P-123 (P123)	2.72	1042	23.0
Pluronic F-127 (F127)	2.81	1511	23.3

**Table 2:** Summary of the electrolyte properties estimated with the proposed rotating ring-disk electrode (RRDE)-based methodology and comparison with the findings reported in the literature.

Disk Material/Electrolyte	υ (cm^2^·s^−1^)	$D_{O_{2}}$ (cm^2^·s^−^^1^)	$D_{O_{2}^{•-}}$ (cm^2^·s^−^^1^)	$C_{O_{2}}$ (mM)	Reference
GC/0.1 M TBAPF_6_, PC	2.6 × 10^−^^2^	1.9 × 10^−^^5^	8.6 × 10^−^^6^	6.1	[6]
GC/0.2 M TBATFSI, PC	2.6 × 10^−^^2^	2.5 × 10^−^^5^	6.8 × 10^−^^6^	4.8	[24]
MnO_2_/C-GC/0.1 M TBAPF_6_, PC	2.6 × 10^−^^2^	1.9 × 10^−^^5^	1.8 × 10^−^^6^	6.1	[6]
M_3_O_4_/C-GC/0.1 M TBAPF_6_, PC	2.6 × 10^−^^2^	1.9 × 10^−^^5^	4.1 × 10^−^^6^	6.1	This work

GC: glassy carbon; TBAPF_6_: tetrabutylammonium hexafluorophosphate; PC: propylene carbonate; TBATFSI: tetrabutylammonium bis(trifluoromethansulfonyl)imide.

**Table 3:** The rate constant for the production of O_2_^•^^−^, *k_f_*, and the PC-electrolyte decomposition rate constant, *k*, for the different sample electrodes.

Disk Materials/Electrolyte (Sample)	*k_f_* (cm·s^−1^)	*k* (s^−1^)
Mn_3_O_4_/C-GC/0.1 M TBAFSI, PC (TX114)	3.7 × 10^−^^2^	3.7
Mn_3_O_4_/C-GC/0.1 M TBAFSI, PC (P123)	2.9 × 10^−^^2^	2.9
Mn_3_O_4_/C-GC/0.1 M TBAFSI, PC (F127)	2.3 × 10^−^^2^	2.3
Super P-GC/0.1 M TBAFSI, PC (SP)	2.1 × 10^−^^2^	2.1
